# Factors associated with signs of congenital syphilis in newborns

**DOI:** 10.1016/j.jped.2024.06.008

**Published:** 2024-07-29

**Authors:** Ana Fátima Braga Rocha, Maria Alix Leite Araújo, Aisha Khizar Yousafzai, Rebeca Gomes de Oliveira, Ana Patrícia Alves da Silva

**Affiliations:** a Universidade de Fortaleza (UNIFOR), Programa de Pós-Graduação em Saúde Coletiva, Fortaleza, CE, Brazil; b Harvard University, Chan School of Public Health, Department of Global Health and Population, Harvard T.H., Boston, MA, United States; c Faculdade Terra Nordeste (FATENE), Fortaleza, CE, Brazil

**Keywords:** Syphilis, Congenital, Signs and symptoms, Prenatal care

## Abstract

**Objective:**

To analyze risk factors (maternal, obstetric and demographic) associated with congenital syphilis and the clinical characteristics of the newborns.

**Method:**

A cross-sectional study carried out in ten public maternity hospitals in Fortaleza, Ceará, Brazil that included cases of live births reported with congenital syphilis in 2015.

**Results:**

469 cases were analyzed; 199 (42.4 %) showed some sign or symptom suggestive of congenital syphilis; of these, 65 (32.7 %) were preterm, 87 (43.7 %) had low birth weight, 116 (58.3 %) had jaundice that required phototherapy, 13 (6.5 %) had hepatomegaly, 10 (5 %) had skin lesions, eight (4.0 %) had splenomegaly and one (0.5 %) had limb pseudoparalysis. Records of other clinical changes were identified in 36 (7.7 %) children. Children whose mothers were not treated or who received a drug other than penicillin and those whose mothers had a VDRL titer ≥ 1:16 at birth were 3.7 and 3.2 times more likely to be born with signs of congenital syphilis (*p* < 0.001- 95 % CI 2.41–5.58; *p* < 0.001 – 95 % CI 2.41–5.58) respectively.

**Conclusions:**

The findings of this study draw attention to the importance of improving the quality of prenatal care and the development of studies aimed at finding alternative drugs for the treatment of syphilis in pregnant women and the prevention of congenital syphilis.

## Introduction

Congenital syphilis (CS) is an infection caused by the etiological agent *Treponema pallidum*, which is transmitted mainly transplacentally from the mother to the fetus.^1^ A study carried out with data from the World Health Organization (WHO) showed that in the global context around 1.36 million pregnant women are infected with syphilis and that in 0.5 million cases, the infection is transmitted to the newborn, causing CS. Approximately 40 % of these cases progress to adverse outcomes such as stillbirth, early fetal death, neonatal deaths, preterm birth, and low birth weight.^2^ For years, the WHO and the Pan American Health Organization (PAHO)^3^ have been proposing the elimination of CS as a public health priority; however, challenges persist to this day.

In Brazil, in 2022 alone, 83,034 cases of syphilis were reported in pregnant women (detection rate of 32.4/1000 live births); 26,468 cases of CS (incidence rate of 10.3/1000 live births), and 200 deaths from CS (mortality rate of 7.8/100,000 live births), with 20.5 % of cases reported in pregnant women and 27.6 % of reported cases of CS occurring in the Northeast region.^4^ In the state of Ceará, northeastern Brazil, 2838 cases of syphilis were reported in 2022 in pregnant women (detection rate of 25.3/1000 live births); with 1488 cases of CS (incidence rate of 13.3/1000 live births), and four deaths from CS (mortality rate of 3.6/100,000 live births).^4^

CS has a wide clinical scope and can result in unfavorable pregnancy outcomes, such as miscarriage, stillbirth, preterm birth, or low birth weight. In live births, attention must be paid to the clinical manifestations of early CS (when they occur up to two years of age), with the main signs being associated with dermatological, bone, ophthalmological, auditory, and neurological findings, in addition to hematological changes.^1^^,^^2^^,^^5^ There is great concern regarding the clinical manifestations of CS in newborns, as they can worsen and have detrimental consequences for the quality of life of these children and thus, they need to be identified as early as possible for appropriate early intervention and management.

Since CS is a preventable condition, the existence of poor CS indicators reflects the quality of care provided to pregnant women. Therefore, the present study aims to analyze risk factors (maternal, obstetric and demographic) associated with CS and the clinical characteristics of the newborns.

## Methods

This cross-sectional study was carried out in Fortaleza, state of Ceará, Brazil, in the ten-county public maternity hospitals, responsible for more than 99 % of CS case notifications in the city. This study is part of a parent study that analyzed the complications, clinical manifestations, and follow-up of reported cases of CS in 2015, a period in which there was a shortage of penicillin in 41 countries. It was approved by the Research Ethics Committee of the University of Fortaleza (UNIFOR), under Opinion number 2110189.

All live births reported with CS in 2015 were included in the study and those who had any co-infection with HIV, hepatitis B and C, toxoplasmosis, rubella, cytomegalovirus, congenital herpes simplex virus infection, and Zika virus infection were excluded, as there was the possibility that it would interfere with the evaluation of CS manifestations. Those cases for which medical records were not available in the maternity wards were also excluded.

To define CS, the following criteria were considered, and established by the Brazilian Ministry of Health.^6^

- Children whose mothers had, during prenatal care or at delivery, reactive (with any titration) non-treponemal tests and reactive treponemal tests, and who had not been treated or had received inadequate treatment.
- Children whose mothers were not diagnosed with syphilis during pregnancy and, given the impossibility of the maternity hospital to perform the treponemal test on them, showed a reactive (with any titration) non-treponemal test at delivery.
- Children whose mothers were not diagnosed with syphilis during pregnancy and, given the impossibility of the maternity hospital to perform the treponemal test on them, showed a reactive treponemal test at delivery.
- Children whose mothers had reactive treponemal tests and non-reactive non-treponemal tests at the time of delivery, without prior treatment.

To define syphilis in pregnant women,^6^ the notification criteria established by the Brazilian Ministry of Health in that year were also used:

- Pregnant woman who had a reactive non-treponemal test at any titer and a reactive treponemal test, regardless of any clinical evidence of syphilis, performed during prenatal care.
- Pregnant woman with a reactive treponemal test and non-reactive or non-reactive non-treponemal test, without record of prior treatment.

Data were collected in 2017 and 2018 from notification forms and medical records of pregnant women and newborns until the moment of discharge from the maternity ward (around ten days of life, as most newborns are hospitalized for CS treatment during that period).

All maternal variables were analyzed as predictive variables: age, level of education, marital status, whether she had any paid work, used any illicit drugs, attended prenatal care appointments, number of prenatal appointments attended, pregnancy trimester at the beginning of prenatal care, number of syphilis tests performed, time of prenatal diagnosis of syphilis, whether the syphilis test was positive during pregnancy (treponemal test or non-treponemal test), treatment received during prenatal care, VDRL test titer at delivery, and whether the partner was also treated.

Maternal VDRL titers at delivery were categorized into ≤1:8 and ≥ 1:16, because high VDRL titers in pregnant women may represent active syphilis, a condition associated with severe CS outcomes.^2^^,^^7^

The outcome variable was the presence of signs suggestive of CS in the newborn. Newborns who had at least one of the early CS signs were considered symptomatic:^1^ preterm birth, low birth weight, hepatomegaly with or without splenomegaly, skin lesions described in the medical record as suggestive of syphilitic rash or syphilitic pemphigus, jaundice requiring phototherapy, serosanguineous rhinitis, and limb pseudoparalysis.

Data were analyzed using the SPSS statistical program, version 23, IBM, USA, and Stata version 10.0 (Stata Corp., USA) using the stepwise multiple logistic regression technique. The descriptive analysis was carried out using the frequency distribution for the categorical variables. Pearson's chi-square test and Fisherʼs exact test were used for the bivariate analysis, considering *p* < 0.05. The adjusted analysis included variables with a *p* value < 0.20, and those with a *p* value < 0.05 remained. The odds ratio (OR) and the 95 % confidence interval were used as a measure of effect size.

## Results

A total of 469 live births with CS were reported in the city of Fortaleza. Table 1 shows the maternal sociodemographic profile and prenatal care. Two hundred and seventy-two (58.0 %) women were aged between 20 and 29 years old, 263 (56.1 %) had a steady partner, 327 (69.7 %) had completed elementary school (≤ 8 years of study), 344 (73.3 %) did not have paid work, and 99 (21.1 %) used illicit drugs (Table 1).

**Table 1:** Sociodemographic profile of mothers of live births reported with congenital syphilis. Fortaleza, 2015. *N* = 469.

Variables	n^a^	%
**Maternal age (in years)**		
≤ 19	95	20.3
20–29	272	58.0
≥ 30	102	21.7
**Marital status**		
With partner	263	56.1
No partner	206	43.9
**Level of schooling**		
Up to complete Elementary School	79	16.8
Incomplete and Complete Junior High	248	52.9
Incomplete High School and more	142	30.3
**Paid work**		
Yes	125	26.7
No	344	73.3
**Illicit drug user**		
Yes	99	21.1
No/Unknown	370	78.9
**Attended prenatal appointments**		
Yes	390	83.2
No	79	16.8
**Number of prenatal appointments** (*n* = 390)		
1	16	4.1
2 to 5	166	42.6
≥ 6	208	53.3
**Start of prenatal (in trimester)** (*n* = 390)		
1st	200	51.3
2nd	148	37.9
3rd	42	10.8
**Number of syphilis tests during the prenatal**^b^ (*n* = 390)		
None	62	15.9
1	251	64.4
2	77	19.7
**Moment of diagnosis of syphilis**		
Prenatal	264	56.3
Delivery	205	43.7
**Syphilis test became positive during pregnancy**^b^ (*n* = 328)		
Yes	77	23.5
No	251	76.5
**Treatment during the prenatal** (*n* = 264)		
Complete^c^	112	42.4
Incomplete^d^	67	25.4
Another drug	7	2.7
Not performed/Unknown	78	29.5
**Partner was treated** (*n* = 173)		
Yes	53	30.6
No/Unknown	120	69.4
**VDRL titer at delivery**		
≤ 1:8	337	71.9
≥ 1:16	132	28.1

a Not all patients underwent prenatal care or all syphilis tests, which is why the “n” of some variables is different.

b Rapid test or VDRL test.

c Three doses of benzathine penicillin G (7.2 million IU).

d One or two doses of benzathine penicillin G (2.4 million IU or 4.8 million IU).

A total of 390 (83.2 %) women attended prenatal care appointments. Of these, 208 (53.3 %) attended six or more appointments, 200 (51.3 %) started prenatal care in the first trimester of pregnancy, and 77 (19.7 %) underwent two syphilis tests throughout the pregnancy. There were no records of other VDRL tests to monitor the cure after treatment (Table 1). During the study period, the rapid test (RT) was in the implementation phase, which is why it was available in some units. The routine test requested for pregnant women was the VDRL and the return of results was available approximately two weeks after the collection.

A total of 264 women (56.3 %) were diagnosed with syphilis during prenatal care and 77 (23.5 %) cases had a positive test during pregnancy, that is, they had a negative result in the first test, and the second test, carried out in the third trimester or at birth, was positive. Only 112 (42.4 %) pregnant women received treatment with three doses of Benzathine penicillin G (BPG; 7.2 million IU). Fifty-three (30.6 %) women had their partner treated with at least one dose of penicillin. Seven (2.7 %) pregnant women received treatment with drugs other than penicillin and 78 (29.5 %) received no treatment or this information was unknown. None of the cases had a record of other VDRL tests to monitor cure after the treatment. At the time of delivery, 132 (28.1 %) women had a titer equal to or greater than 1:16 (Table 1).

Table 2 describes the signs of CS observed in newborns at delivery. One hundred and ninety-nine (42.4 %) had some sign suggestive of CS; of these, 65 newborns (32.7 %) were preterm, 87 (43.7 %) had low birth weight, 116 (58.3 %) had jaundice requiring phototherapy, 13 (6.5 %) had hepatomegaly, 10 (5 %) had skin lesions, eight (4.0 %) had splenomegaly, and one (0.5 %) had limb pseudoparalysis. Records of other clinical alterations were identified in 36 (7.7 %) children (including respiratory, cardiocirculatory, genitourinary, bone, neurological, and ophthalmological problems).

**Table 2:** Presence of signs in newborns reported with congenital syphilis. Fortaleza, 2015. *N* = 469.

Variables	n	%
**Clinical manifestations related to CS** (*n* = 199)^a^		
Jaundice requiring phototherapy	116	58.3
Low birth weight	87	43.7
Preterm birth	65	32.7
Hepatomegaly	13	6.5
Skin lesions	10	5.0
Splenomegaly	8	4.0
Limb pseudoparalysis	1	0.5
**Other clinical alterations** (*n* = 36)^a^		
Respiratory	19	52.8
Cardiocirculatory	10	27.8
Genitourinary	4	11.1
Bone^b^	4	11.1
Neurological^c^	2	5.5
Ophthalmological	1	2.8

a Some newborns had more than one clinical manifestation and/or other alterations, which is why the total exceeds 100 %.

b Bone alterations without performing a radiography: two cases described as “characteristic face: Olympian forehead, low ear implantation and micrognathia”, one case with “saddle nose” and another described as “probable fracture with swelling in the lower limbs and crying when handled, requiring investigation due to congenital syphilis ”.

c Neurological alterations described in the medical files as global developmental delay.

The bivariate analysis showed a statistically significant association between the presence of signs of early CS in newborns whose mothers did not attend prenatal care (*p* < 0.001), attended fewer than six appointments (*p* = 0.010), did not undergo any VDRL tests (*p* = 0.031), mothers whose syphilis test became positive during pregnancy (*p* = 0.003), were diagnosed with syphilis at the time of delivery (*p* < 0.001), were not treated during prenatal care or the treatment was carried out with a drug other than penicillin (*p* = 0.008), the partner was not treated (*p* = 0.001), and had a VDRL titer ≥ 1:16 at delivery (*p* < 0.001; Table 3).

**Table 3:** Signs of congenital syphilis in newborns in relation to prenatal care. Fortaleza, Ceará, 2015. *N* = 469.

	Newborn				
	Asymptomatic		Symptomatic		
Variables	n^a^	%	n^a^	%	Valor *p*
**Attended prenatal** (*n* = 456)					<0.001*
Yes	232	61.2	147	38.8	
No	25	32.5	52	67.5	
**Number of appointments** (*n* = 379)					0.010*
< 6 appointments	95	54.3	80	45.7	
≥ 6 appointments	137	67.2	67	32.8	
**Start of the prenatal (in trimester)** (*n* = 379)					0.328
1st	124	63.6	71	36.4	
2nd– 3rd	108	58.7	76	41.3	
**Number of syphilis tests performed in the prenatal**^b^ (*n* = 379)					0.031*
None	27	48.2	29	51.8	
At least one	205	63.5	118	36.5	
**Syphilis test became positive during the pregnancy**^b^ (*n* = 323)					0.003*
Yes	36	48.6	38	51.4	
No	169	67.9	80	32.1	
**Moment of the diagnosis of syphilis in pregnant woman** (*n* = 456)					<0.001*
Prenatal	179	68.3	83	31.7	
Delivery	78	40.2	116	59.8	
**Treatment of the pregnant woman** (*n* = 456)					<0.001*
Benzathine penicillin G^c^	131	73.6	47	26.4	
Another dug/Not treated^d^	126	45.3	152	54.7	
**Was the partner treated?** (*n* = 173)					0.001*
Yes	46	86.8	07	13.2	
No	73	60.8	47	39.2	
**VDRL titer at delivery** (*n* = 456)					<0.001*
≤ 1:8	210	62.7	125	37.3	
≥ 1:16	47	38.8	74	61.2	

⁎ p<0.05. Fisher's exact test.

a Not all patients underwent prenatal care or all syphilis tests, which is why the “n” of some variables is different.

b Rapid test or VDRL test.

c At least one dose (2.4 to 7.2 million IU).

d Pregnant women who did not undergo treatment whether or not they attended prenatal care.

The adjusted multivariate logistic regression analysis showed that children whose mothers were not treated or received another drug other than penicillin and those whose mothers had a VDRL titer ≥ 1:16 at delivery were 3.7 and 3.2 times more likely to be born with signs of CS (*p* < 0.001 - 95 % CI 2.41–5.58; *p* < 0.001 – 95 % CI 2.41–5.58) respectively (Table 4).

**Table 4:** Multivariate logistic regression analysis, adjusted and unadjusted, of the presence of signs of congenital syphilis at delivery associated with prenatal care. Fortaleza, Ceará, 2015. *N* = 469.

	Presence of signs in the newborn							
			Non-adjusted			Adjusted		
Variables	n	%	OR	(95 %CI)	*p*-value	OR	(95 %CI)	*p*-value
Did not attend prenatal (*n* = 456)	52	67.5	3.2	(1.95–5.52)	<0.001	–	–	–
Fewer than 6 prenatal appointments (*n* = 379)	80	45.7	1.7	(1.13–2.61)	0.010	–	–	–
No tests performed during prenatal (*n* = 379)	29	51.8	1.8	(1.05–3.30)	0.031	–	–	–
Test became positive during pregnancy (*n* = 323)	38	51.4	2.2	(1.31–3.78)	0.003	–	–	–
Diagnosis of the pregnant woman during delivery (*n* = 465)	116	59.8	3.2	(2.17–4.72)	<0.001	–	–	–
Not treated with penicillin or without treatment during pregnancy (*n* = 456)	152	54.7	3.3	(2.23–5.05)	<0.001	3.7	(2.41–5.58)	<0.001
Partner not treated during pregnancy (*n* = 173)	47	39.2	4.2	(1.76–10.15)	0.001	–	–	–
VDRL titer at delivery ≥ 1:16 (*n* = 456)	74	61.2	2.6	(1.72–4.05)	<0.001	3.2	(2.01–4.85)	<0.001

## Discussion

This study demonstrated that the presence of signs of early CS is related to missed opportunities, especially in relation to testing for syphilis and treatment of pregnant women during prenatal care. Mothers with syphilis live in a situation of greater social vulnerability. They are mostly young individuals, of reproductive age, with a low level of education and no income,^8^ which makes CS control even more challenging. People in vulnerable situations often experience greater difficulties in accessing health services; thus, they require different and potentially more targeted and responsive care from health services and social institutions.^9^

The recommended strategies for the care of pregnant women aimed at preventing CS involve early recruitment to start prenatal care, timely testing and appropriate treatment, including their sexual partners, in addition to the active search for pregnant women who interrupted the prenatal care.^1^

The RT can contribute greatly to improving testing and treatment coverage of pregnant women. It so happens that, in Brazil and in some other countries worldwide, the RT is not yet effectively implemented in the first prenatal consultation,^10^, ^11^, ^12^ representing missed opportunities for the prevention of CS. It is important that basic health units organize pregnant women's demand for care, to consider the time needed to perform the RT at the time of the consultation.

Most newborns who showed signs of CS were children of pregnant women whose first test results for syphilis were negative (i.e., they became positive during pregnancy) and those who had high titers in the VDRL test at the time of delivery. It is likely that these mothers became infected during pregnancy and had an active infection, a situation that can result in severe outcomes for the newborn.^13^^,^^14^ This fact is corroborated by the large proportion of pregnant women who were not tested in the third trimester of pregnancy.

It was observed that the majority of pregnant women with syphilis did not receive adequate treatment, a condition that may be related both to the problem of penicillin shortage experienced in Brazil^15^ during this study period, and the refusal of professionals to treat pregnant women in some primary care units for fear of an anaphylactic reaction.^16^ It is known that the harm resulting from not administering penicillin to pregnant women is much more severe than the risk of an anaphylactic reaction.^17^

This study showed that the children of mothers treated with drugs other than penicillin and those whose mothers did not receive any treatment were the ones with the most signs at delivery. Benzathine penicillin G is the only recommended treatment to prevent mother-to-child transmission of syphilis;^1^^,^^3^^,^^15^^,^^17^ for this reason, the penicillin shortage period was an event that further compounded prenatal care challenges, with negative effects on CS control.

It is also worth highlighting the high percentage of pregnant women who did not have their sexual partners treated. At the time when these pregnant women were notified, and the partner was not treated, the pregnant woman was considered to be inadequately treated.^6^ Currently, this criterion is no longer considered^4^; however, the treatment of the partner(s) remains essential to prevent reinfection in pregnant women and the primary care service needs to implement strategies to engage these partners, such as the Partner Prenatal Care.^18^ It is on these occasions that participation in appointments, childbirth and postpartum is encouraged, in addition to the possibility of providing health educational guidance, counseling, updating the partner's vaccination card, and offering routine examinations and rapid tests for sexually transmitted infections.^18^

The most frequent clinical manifestations of CS identified in this study were jaundice, low birth weight, and preterm birth. It is necessary to consider that jaundice is a non-specific sign, one of the most prevalent among newborns,^19^ especially among those with extremely low birth weight (< 1.500 kg) and preterm newborns.^20^

Regarding low birth weight and prematurity, the proportion of these findings was higher than those found at the national level^21^ and global estimates,^2^ which may be related to the context of the syphilis epidemic in the region where these newborns were born. Data from the *Nascer no Brasil* study revealed that in 2015, the year of birth and notification of these newborns, the highest rate of vertical transmission of syphilis occurred in the Northeast region^21^ and official statistics from the Ministry of Health show that this region has one of the highest CS incidence rates in the country.^4^

In relation to other clinical manifestations characteristic of early CS, such as hepatomegaly, splenomegaly and skin lesions, the values found were lower than those of the Porto Alegre cohort.^5^ However, it is necessary to consider that the cases analyzed in that cohort did not include the children of adequately treated pregnant women when lower symptom occurrence is expected.

Moreover, the possibility of underreporting of these symptoms cannot be ruled out. The reality of overcrowding experienced in maternity wards of the Brazilian Unified Health System^22^ can limit the time for adequate recording in medical files. It is also possible that professionals need training to better identify the presence of clinical manifestations of CS, considering that this infection has been neglected for many years and that the signs of CS are nonspecific, making it important to perform a differential diagnosis.

This study's limitation is the fact that it analyzed secondary data, making the analysis subject to the lack of records or low-quality ones. This condition hindered the identification of other maternal or gestational conditions that may cause signs suggestive of CS in newborns. It is recognized that the analyzed signs are nonspecific ones; however, all newborns were reported with CS and these signs are recognized in the literature as associated with this condition.^1^ It is worth noting that cases of co-infection were excluded from the analysis.

The present study showed that the presence of signs of CS in the newborn is related to the lack of treatment or treatment of the pregnant woman with a drug other than penicillin, as well as the presence of high VDRL titers at the time of delivery. These findings draw attention to the importance of improving the quality of prenatal care and the development of studies to find alternative drugs for the treatment of syphilis in pregnant women and the prevention of CS, considering the possibility of new episodes of penicillin shortages.^23^

Furthermore, it is recommended that, in cases of newborns born to untreated or inadequately treated mothers, a strict investigation be carried out with a thorough physical examination, ensuring that the recommended tests are carried out, with adequate outpatient follow-up after discharge from the maternity ward.^1^

## Financial support

This work received funding from the University of Fortaleza (UNIFOR) through announcement 60/2022 of the Research Team Support Program.

## CRediT authorship contribution statement

**Ana Fátima Braga Rocha:** Methodology, Data curation, Formal analysis, Writing – original draft. **Maria Alix Leite Araújo:** Methodology, Data curation, Formal analysis, Writing – review & editing. **Aisha Khizar Yousafzai:** Writing – review & editing. **Rebeca Gomes de Oliveira:** Methodology, Data curation, Formal analysis. **Ana Patrícia Alves da Silva:** Data curation, Writing – review & editing.

## Conflicts of interest

The authors declare no conflicts of interest.
